# Mechanical stretch: physiological and pathological implications for human vascular endothelial cells

**DOI:** 10.1186/s13221-015-0033-z

**Published:** 2015-09-18

**Authors:** Nurul F. Jufri, Abidali Mohamedali, Alberto Avolio, Mark S. Baker

**Affiliations:** Department of Biomedical Sciences, Faculty of Medicine and Health Sciences, F10A, 2 Technology Place, Macquarie University, Sydney, NSW 2109 Australia; Department of Chemistry & Biomolecular Sciences, F7B Building Research Park Drive, Macquarie University, Sydney, NSW 2109 Australia

## Abstract

Vascular endothelial cells are subjected to hemodynamic forces such as mechanical stretch due to the pulsatile nature of blood flow. Mechanical stretch of different intensities is detected by mechanoreceptors on the cell surface which enables the conversion of external mechanical stimuli to biochemical signals in the cell, activating downstream signaling pathways. This activation may vary depending on whether the cell is exposed to physiological or pathological stretch intensities. Substantial stretch associated with normal physiological functioning is important in maintaining vascular homeostasis as it is involved in the regulation of cell structure, vascular angiogenesis, proliferation and control of vascular tone. However, the elevated pressure that occurs with hypertension exposes cells to excessive mechanical load, and this may lead to pathological consequences through the formation of reactive oxygen species, inflammation and/or apoptosis. These processes are activated by downstream signaling through various pathways that determine the fate of cells. Identification of the proteins involved in these processes may help elucidate novel mechanisms involved in vascular disease associated with pathological mechanical stretch and could provide new insight into therapeutic strategies aimed at countering the mechanisms’ negative effects.

## Introduction

Blood vessels consist of three primary layers: the tunica intima, the tunica media and the tunica adventitia. The tunica intima is the innermost layer that contains the endothelium (endothelial cell (EC) layers) that provides a smooth surface for blood flow, whereas the tunica media contains thick layers of elastin, collagen and smooth muscle cells (SMCs) for vascular dilation or constriction. The outermost layer, the tunica adventitia, is composed of a mixture of connective tissue, collagen and elastic fibers and is used for arterial support. Hemodynamic forces, such as shear and tensile stress, continuously act upon blood vessels due to the pumping motion of the heart. Specifically, shear stress arises from the friction of the blood flow with the endothelial layer, whereas tensile stress primarily acts upon the medial layers and is due to the pulsatile nature of blood pumped from the heart.

Mechanical stretch enables vascular maintenance through proliferation, angiogenesis, the formation of reactive oxygen species, control of vascular tone and vascular remodeling [1–6]. However, the excessive mechanical stretch that occurs during hypertension has been shown to be detrimental as it perturbs these processes and causes inappropriate cellular responses that can lead to cardiovascular abnormalities [7]. As such, mechanical stretch has been modeled *in vitro* by regulating stretch intensity to simulate physiological and pathological stretch magnitudes (the percentage of the cell elongation from the cell’s original dimensions). Low magnitude stretches of 5–10 % are categorized as physiological stretch, whereas high magnitude stretches of 20 % and above are considered pathological stretch and are thought to simulate what is proposed to occur during hypertension [8]. The differences in stretch intensity may activate different downstream signaling pathways that determine the cells’ functional, biological and phenotypic features.

Previous studies have focused on the effect of shear stress and its pathological implications on EC. However, the effect of tensile stretch (specifically on human vascular ECs), has not been studied in depth [9–12]. For this reason, this review will focus on the current research in mechanotransduction specifically as it relates to vascular ECs. There will be a particular emphasis on receptors involved in sensing mechanical stretch; the signal transduction pathways involved that result in extracellular matrix (ECM) remodeling, angiogenesis, cell proliferation, vascular tone homeostasis, reactive oxygen species formation, inflammation and apoptosis (Table 1). In addition, the review will attempt to relate how these functions are thought to be associated with the development of disease. Finally, we will briefly discuss the direction of future research in this field.

**Table 1:** Mechanical stretch induces various biological processes in endothelial cells

	Cell type	Stretch intensity	Observation/Measurement	Biological process	Reference
1	HUVEC	10 %	↑ actin	Morphology	Yoshigi *et al.* 2003 [29]
2	HUVEC	10 %	Cells oriented 65 ° to stretch direction	Morphology	Barron *et al.* 2007 [32]
3	HUVEC	0–110 %	Cells oriented 47.8 ° at 100 %	Morphology	Takemasa *et al.* 1998 [27]
4	HAEC	10 %	Cells oriented at 70/90 °	Morphology	Wang *et al.* 2001 [34]
5	HUVEC	0–25 %	Cells oriented at 60–70 ° at 10–15 % stretch	Morphology	Haghighipour *et al.* 2010 [94]
6	HUVEC	10 %	Perpendicular cell’s orientation	Morphology	Moretti *et al.* 2004 [31]
7	HUVEC	20 %	Paxillin needed for initial cell orientation	Morphology	Huang *et al.* 2012 [30]
8	BAEC	1–10 %	Rho proteins for perpendicular alignment	Morphology	Kaunas *et al.* 2005 [35]
9	BAEC	1–10 %	↑ JNK (2.6-fold) at 30 min	Morphology	Kaunas *et al.* 2006 [36]
10	HUVEC	120 %	↑ CAMP (3-fold)	Morphology	Yamada *et al.* 2000 [96]
11	HUVEC	Local stretch by microneedle	↑ Src homology 2-containing tyrosine phosphatase	Morphology	Ueki *et al.* 2009 [25]
12	BAEC	5–30 %	↑ Hsp 25 (relative activity 40 %)	Morphology	Luo *et al.* 2007 [38]
			↑ Hsp 70 (relative activity 60 %)		
13	BAEC	10 %	↑ JNK (5-fold)	Morphology	Hsu *et al.* 2010 [37]
			↑ ERK (4-fold)		
			↑ p38 (4-fold)		
14	HUVEC	120 %	↑ Ca^2+^	Calcium influx	Naruse *et al.* 1998 [14]
15	BCE	10/15 %	↑ Ca^2+^ (2-fold) via transient receptor potential vanilloid 4	Calcium influx	Thodeti *et al.* 2009 [13]
16	bEND	20/35/55 %	↑ Ca^2+^ via transient receptor potential channels	Calcium influx	Berrout *et al.* 2012 [16]
17	HUVEC	20 %	↑ c-src (3.2-fold) at 15 min	Mechanotransduction	Naruse *et al.* 1998 [97]
18	HUVEC	20 %	↑ pp125^FAK^	Mechanotransduction	Naruse *et al.* 1998 [98]
19	BAEC	10 %	↑ p21ras (24.7 % ratio) at 1 min	Mechanotransduction	Ikeda *et al.* 1999 [22]
20	HUVEC	20 %	↑ tyrosine phosphorylation (>2000 arbitrary unit)	Mechanotransduction	Katanosaka *et al.* 2008 [20]
21	BAEC	5–25 %	↑ ERK at 15 mins	Mechanotransduction	Shi *et al.* 2007 [23]
22	HUVEC	120 %	↑ integrin beta-3 (171 %) at 4 h	Adhesive	Suzuki *et al.* 1997 [17]
23	HAEC	5–20 %	↑ Akt phosphorylation at 5 %, 10 min (6000 arbitrary unit)	Apoptosis	Kou *et al.* 2009 [77]
			↑ Akt phosphorylation at 20 %, 30 min (1000 arbitrary unit)		
24	BAEC	6–10/20 %	↑ Akt phosphorylation and ↑ Bad phosphorylation in presence of TNFα at 6 %	Apoptosis	Liu *et al.* 2003 [45]
25	BAEC	10 %	↑ Akt phosphorylation at Ser 473 at 30–60 mins	Apoptosis	Nishimura *et al.* 2006 [78]
26	BAEC	10 %	↑ S6K phosphorylation (1.5-fold) at 30 mins	Proliferation	Li and Sumpio 2005 [1]
27	BAEC	20 %	↑ Rac1 (5-fold)	Proliferation	Liu *et al.* 2007 [56]
28	HUVEC	20 %	↑ c-Myc (2–3-fold) at 1–2 h	Proliferation	Hurley *et al.* 2010 [57]
29	Vein graft	15 %	↑ Egr-1 (5.5-fold) at 90 mins	Proliferation	Zhang *et al.* 2013 [58]
	HUVEC	20 %	↑ MMP-2 (3.7-fold) at 18 h	Extracellular matrix	Wang *et al.* 2003 [44]
			↑ MMP-14 (3-fold) at 18 h		
30	BAEC	5 %	↑ MMP-2 (8-fold) at 8 h	Extracellular matrix	von Offenberg Sweeney *et al.* 2004 [43]
31	BCEC	0–28 %	↑ pro MMP-2	Extracellular matrix	Shukla *et al.* 2004 [99]
32	HUVEC	15 %	↑ MCP-1 (200 %) at 6 h	Inflammation	Demicheva *et al.* 2008 [100]
33	HUVEC	125/150 %	↑ IL-6 (3-fold) at 90 min	Inflammation	Kobayashi *et al.* 2003 [75]
34	HUVEC	0–10 %	↑ COX-2 (2.5-fold) at 3 h	Inflammation	Zhao *et al.* 2009 [101]
			↑ thromboxane A_2_ synthase (150 %)		
35	HUVEC	120–150 %	↑ von Willebrand factor to 5 mU/L at 60 min	Inflammation	Xiong *et al.* 2013 [74]
36	HUVEC	6–15 %	↑ IL-8 (2.6-fold)	Inflammation	Okada *et al.* 1998 [80]
			↑ MCP-1 (2.8-fold)		
37	HUVEC	12–25 %	↓ MCP-1 by exposure of nitric oxide donor	Inflammation	Wung *et al.* 2001 [102]
38	BAEC	0–10 %	MMP-9 silencing block migration and tube formation	Angiogenesis	von Offenberg Sweeney *et al.* 2005 [55]
39	BAEC	10 %	Endothelial cord aligning to 67.5–90°	Angiogenesis	Joung *et al.* 2006 [2]
40	HUVEC	6–13 %	↑ Ang-2 (4.8-fold)	Angiogenesis	Yung *et al.* 2009 [54]
			PDGF-ββ (↑ 5.0-fold)		
41	CMEC	10 %	↑ VEGF (1.4-fold)	Angiogenesis	Zheng *et al.* 2001 [51]
42	CMEC	10 %	↑ VEGF-R2 (3.2-fold)	Angiogenesis	Zheng *et al.* 2008 [48]
			↑ Tie-2 (1.8-fold)		
43	BAEC	10/20 %	↑ angiogenesis	Angiogenesis	Wilkins *et al.* 2014 [52]
44	HUVEC	15 %	↑ Flk-1 (1.7-fold)	Angiogenesis	Zheng *et al.* 2004 [47]
			↑ Tie-1 (2-fold)		
			↑ Tie-2 (1.9-fold)		
45	HUVEC	10 %	↑ Alpha smooth muscle actin (1.6-fold)	Transdifferentiation	Shoajei *et al.* 2014 [84]
			↑ Smooth muscle myosin heavy chain (1.3-fold)		
46	HUVEC	10 %	↑ Alpha smooth muscle actin (165 %)	Transdifferentiation	Cevallos *et al.* 2006 [83]
			↑ Caldesmon-1 (443 %)		
			↑ Smooth muscle myosin heavy chain (205 %)		
			↑ Calponin-1 (174 %)		
47	HUVEC	10/20 %	↑ Young’s modulus of elasticity	Stiffening	Hatami *et al.* 2013 [82]
48	BAEC	120 %	↑ eNOS phosphorylation (1.8 arbitrary unit)	Vascular tone	Takeda *et al.* 2006 [62]
			↑ NO (1.4-fold)		
49	HUVEC	20–50 %	↑ eNOS (7-fold) at 50 %	Vascular tone	Hu *et al.* 2013 [5]
50	HUVEC	25 %	↑ Et-1 (2.3-fold)	Vascular tone	Cheng *et al.* 2001 [4]
51	HUVEC	10 %	↑ Et-1 (1.6-fold)	Vascular tone	Toda *et al.* 2008 [64]
52	HUVEC	6 %	↑ 8,9-epoxyeicosatrienoic acid (EET) (4–8-fold)	Vascular tone	Fisslthaler *et al.* 2001 [63]
53	HUVEC	100–250 %	↑ glutathione peroxidase to 200 %	ROS	Wagner *et al.* 2009 [71]
54	HAEC	8–20 %	↑ p66^Shc^ (150–200-fold)	ROS	Spescha *et al.* 2014 [70]
55	HUVEC	5–12 %	↓ Nox4 (40 %) 12 %, 24 h	ROS	Goettsch *et al.* 2009 [67]
			↑ eNOS (3-fold) at 12 %, 24 h		
			↑ NO (2 uM) at 5 %, 24 h		
56	HUVEC	25 %	↑ ROS (221 %), 6 h	ROS	Ali *et al.* 2004 [68]
			↑ VCAM-1		
57	BAEC	25 %	↑ FAK phosphorylation	ROS	Ali *et al.* 2006 [66]

*HUVECs* Human umbilical vascular endothelial cells, *HAECs* Human aortic endothelial cells, *BAECs* Bovine arterial endothelial cells, *BCE* Bovine capillary endothelial cells, *bEND* Brain microvessel endothelial cells, *BCECs* Brain capillary endothelial cells, *CMECs* Coronary microvascular endothelial cells

### Mechanical stretch receptors induce signal transduction

Mechanical stretch generates a cascade of biochemical signaling processes in ECs. The fundamental paradigm is that mechanoreceptors on the plasma membrane of ECs, through a series of signaling pathways, induce gene expression and protein synthesis to promote or ablate processes such as angiogenesis, proliferation, inflammation, apoptosis, vascular tone and cell survival.

Biochemically, mechanotransduction of stretch is detected by three known mechanoreceptor proteins that are distributed throughout the cell: stretch activated (SA) channel, integrin proteins and the platelet endothelial cell adhesion molecule-1 (PECAM). The SA channel is located on the plasma membrane and has been shown to participate in calcium (Ca^2+^) influx in response to stretch that later initiates PI3K activation mediated by Rho and Rho-associated kinase (ROCK) for cellular orientation [13, 14]. Studies on the SA channel have determined that the specific ion channel known as the transient receptor potential (TRP) is responsible for the Ca^2+^ influx [13]. The transient receptor potential vanilloid channel 4 (TRPV4) is found to be highly expressed in ECs. Meanwhile, brain ECs specifically exhibit transient receptor potential classical 1 (TRPC1) and transient receptor potential polycystin 2 (TRPP2) [15]. Knockdown of TRPP2 has been found to inhibit the Ca^2+^ influx, and this leads to disruption of blood–brain barrier integrity and to edema [16].

Integrins are the second known type of mechanoreceptors that act by transmitting stretch signals from the ECM into the cell. Integrins are transmembrane heterodimeric glycoproteins consisting of one of 8 different α and one of 18 different β subunits. They attach the cell to the ECM and to proteins located within the matrix (e.g., latent TGFβ1 for ITGB6). Stretch-exposed HUVECs express higher levels of the endothelial cell integrin αVβ_3_ through P13K activation, suggesting enhanced adhesiveness of the cells to RGD (tripeptide of L-arginine, glycine, and L-aspartic acid)-containing ECM substrates such as fibronectin [17, 18]. As integrin-ECM binding is increased, it stimulates an increased level of Ca^2+^ influx that is associated with promoting phosphorylation of focal adhesion kinase (FAK) and Src family kinases proximal to the inner surfaces of the integrin. FAK is one of the components of the focal adhesion complex which is composed of a group of proteins (i.e., zyxin, vinculin, talin, paxillin and actinin) that function together to connect the ECM and integrins to the cytoskeleton at the plasma membrane [19]. In addition, mechanical stretch induces Src tyrosine kinase activation of molecules localized to the focal adhesion (FA), and this appears to be central to signal transduction pathways and changes actin organization in HUVECs [20].

The third class of proteins that act as a mechanoreceptors is the platelet endothelial cell adhesion molecule-1 (PECAM-1), also known as CD31. It is a cell adhesion molecule that is abundantly expressed in ECs, especially in regions of cell-to-cell contact [21]. It is suggested that the application of a specific force generates EC deformation, and PECAM-1 is able to sense this change from the neighboring cells through PECAM-1 tyrosine phosphorylation. This is then followed by activation of the extracellular signal-related kinase 1/2 (ERK1/2) signaling cascade via P21ras and Raf-1 [21–23]. In addition, PECAM-1 phosphorylation initiates SHP-2 binding to activate MAPK and ERK1/2 pathways that promote cellular reorientation [24, 25]. Expression of these mechanoreceptor proteins across the EC indicates that sensing the force is a crucial initial step to activate mechanotransduction.

### Morphology and structural changes induced by mechanical stretch

**Fig. 1:**
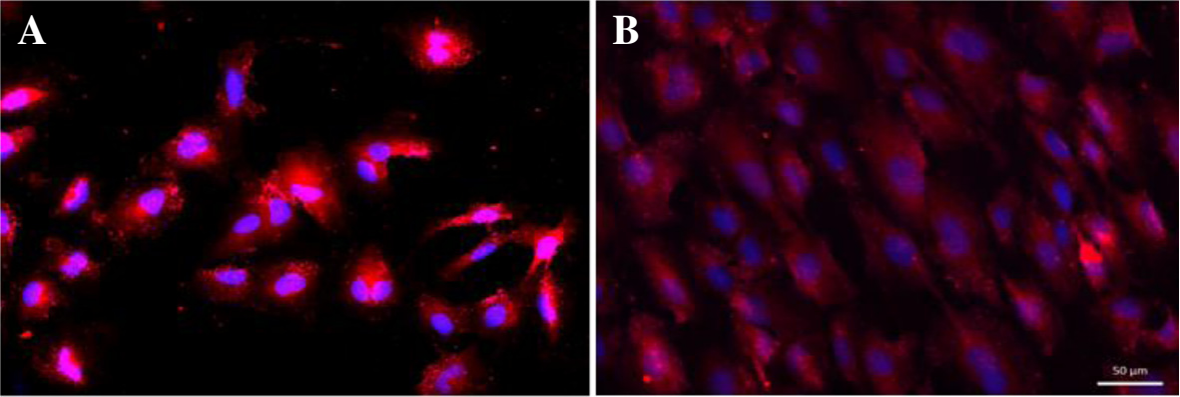
Morphological change of human cerebral microvascular endothelial cells (HCMECs). The HCMECs were stained with Alexa 594 (*red*) for actin, and the nucleus was stained by DAPI (*blue*). **a** HCMECs that were not exposed to stretch were rounded in shape. **b** HCMECs that were exposed to 18 h cyclic stretch became elongated in shape

The morphological and structural changes in cells are primarily determined by the cytoskeleton and focal adhesion complexes. One of the distinct responses of ECs exposed to stretch is the emergence of a bundle of 10–30 actin filaments, known as stress fibers, which contribute to resistance against the applied stress and transmit mechanotransduction in non-muscle cells [26–28]. ECs cultured under static conditions exhibit a polygonal shape and are randomly orientated. However, two main morphological changes are observed when mechanical stretch is applied to ECs. First, cells become elongated and second, become slanted to a particular angle usually perpendicular to the stretch direction due to stress fiber reorientation (Fig. 1) [14, 29–32]. Previous studies have determined that the perpendicular stress fibers’ orientation serves to maintain the cell structure for minimizing alterations in intracellular strain by bearing less tension [33, 34]. This orientation is mediated by the activation of the Rho pathway, as inhibition of Rho perturbs the perpendicular orientation of stress fibers [35].

The perpendicular orientation of early phase ECs is mediated by paxillin, one of the signaling structural scaffold proteins found in the FA complex [30]. Knockdown of paxillin abolishes the perpendicular orientation of stretched HUVECs, suggesting it plays a pivotal role in aligning stress fibers during stretch [30]. Equally, stretching increases JNK and ERK phosphorylation during the early stages of stress fiber orientation, and these levels subside after the stress fiber is oriented perpendicular to the stretch direction [36, 37]. In addition, heat shock protein 70 (HSP70) expression has also been shown to be increased by stretch and its inhibition shown to inhibit EC stress fiber formation [38]. Thus, these intracellular signals are suggestive of complex processes involved in the regulation of stress fibers in determining EC morphology when they are subjected to mechanical stretch.

### Extracellular matrix remodeling by mechanical stretch

The ECM comprises a mixture of molecules, such as collagen, elastin, proteoglycans, laminin and fibronectin that provide structural support, adhesion sites and transmission of biochemical signals to surrounding cells [39]. Synthesis and degradation of ECM is an essential part of the vascular remodeling process for homeostasis and during physiological and pathological responses. Zinc-dependent endopeptidases from the matrix metalloproteinase (MMP) protease family can induce the breakdown of ECM if the zymogen MMPs are activated physiologically [40–42]. MMPs contribute to vascular remodeling through vascular adaptation, angiogenesis and repair during physiological stretch. Physiological stretch increases MMP-2 expression in bovine arterial endothelial cells (BAEC), and this is thought to be mediated by the Gβγ/p38 and PTK/Shc/ERK pathways [43]. By contrast, pathological stretch increases both MMP-2 and MMP-14 in HUVECs, and this was shown to be mediated through the TNF-α and JNK pathways [44, 45]. MMP activity during pathological stretch is thought to contribute to atherosclerosis as it facilitates the migration of vascular smooth muscle cells into the intima layer where further proliferation contributes to plaque formation [46].

### Physiological stretch induces angiogenesis

Vascular ECs are known to play a major role in angiogenesis as they are involved in vessel cord formation, sprouting, migration and tube formation, and this appears to be facilitated by a series of chemical stimuli (Table 1). Several processes involved in angiogenesis have been associated with physiological stretch. For example, physiological stretch has been found to upregulate key tyrosine kinase receptors such as Flk-1, Tie-2 and Tie-1 in both HUVECs and RCMECs [47, 48]. These receptors are sensitive to growth factors and act to induce the formation of new blood vessel.

In addition, stretch stimulates the secretion of angiogenic factors that circulate in a paracrine or autocrine manner in the vascular system [49, 47]. Physiological stretch has been reported to increase the secretion of vascular endothelial growth factor (VEGF) and the expression of its receptor, VEGF-R2 (Flk-1) [49]. Both of these are key proteins needed for cell proliferation and tube formation during HUVEC angiogenesis [50, 51]. In addition, basic fibroblast growth factor (bFGF) was also increased and found to promote sprouting during angiogenesis when ECs were subjected to stretch [52]. bFGF may be released at the initial state of angiogenesis before being replaced by VEGF to complete the angiogenesis process [53]. Furthermore, physiological stretch was found to activate endogenous biochemical molecules such as angiopoietin-2 and platelet derived growth factor ββ (PDGF-ββ) that may be involved in endothelial cell migration and sprout formation [54]. EC migration and tube formation were also increased during stretch due to the activation of G_i_ protein subunits and increased GTPase activity which facilitates angiogenesis [55]. Taken together, these results show that physiological stretch is intimately involved in evoking vasculature angiogenic processes across the vascular system.

### Mechanical stretch stimulates EC proliferation

Cell proliferation is a fundamental process for replacing old and damaged cells and represents an important part of tissue homeostasis and stretch is thought to influence this biological function (Table 1). Exposure to physiological stretch in BAECs was found to induce cell proliferation, mediated by the P13K-dependent S6K mTOR-4E-BP1 pathway [1]. The mammalian target of rapamycin (mTOR) is an important key translational pathway that regulates cell cycle, proliferation and growth. In addition, cell-to-cell adhesion is required for ECs to proliferate during stretch. This cell-to-cell adhesion is principally mediated by cadherins that transduce mechanical forces through Rac1 activation [56]. This may limit stretch-mediated EC proliferation as it occurs only in the presence of adjacent cells and serves as a mechanism to prevent ECs from displaying elements of invasive behavior and/or excessive proliferation [56]. However, uncontrolled proliferation of ECs has been observed in pathological stretch as the expression of the oncogene c-Myc was upregulated in HUVEC [57]. This could be a major contributor to vascular disease as it could lead to the intimal thickening that increases vascular resistance and blood pressure. In addition, the observation that early growth response protein-1 (Egr-1) promotes proliferation during stretch in vein graft models supports the suggestion that pathological stretch plays a role in restenosis [58]. Thus, future strategies aimed at targeting these proteins may be of therapeutic value for controlling cell proliferation that originates from hypertension.

### Expression of vasoconstrictors and vasodilators during stretch

Blood vessels depend on numerous vasodilating and vasoconstricting protein factors to regulate vascular tone through the homeostatic balancing of blood pressure (Table 1). Endothelin 1 (ET-1) is a potent vasoconstrictor produced by vascular ECs. The endothelium-derived hyperpolarizing factor (EDHF) induced by epoxyeicosatrienoic acid (EET) generated by the cytochrome P450 (CYP) epoxygenase enzyme subfamily is another vasoconstrictor that functions to increase blood pressure [59, 60]. On the other hand nitric oxide (NO) plays an important role in vasodilation and is generated from the conversion of L-arginine to L-citrulline by phosphorylated endothelial nitric oxide synthase (eNOS) [61]. The synthesis of eNOS is controlled by stretch, and its production is dependent on Ca^2+^ influx. Specifically, a decrease of Ca^2+^ elicited by an inhibitor of the SA channel was shown to inhibit eNOS phosphorylation [62]. Physiological stretch was found to increase ET-1 mRNA levels in HUVECs, whereas EET and CYP 2C mRNA expression for the generation of EDHF was increased in the coronary artery of ECs [63]. Pathological stretch was found to increase ET-1 in HUVECs [4, 64] whereas eNOS and NO were increased in BAECs and HUVECs [5, 62]. Several mechanisms have been proposed for the regulation of NO expression, such as an increase of Ca^2+^ concentration via the stretch-activated channel at the early phase of stretch followed by eNOS phosphorylation via the PKA pathway and activation of the P13K-Akt/PKB pathway in the late stage of stretch [5, 62, 65]. NO has anti-atherogenic properties, as it inhibits transcription factors that regulate expression of pro-atherogenic or pro-inflammatory genes. However, the balance of NO might be altered in pathological stretch as the ROS levels are often elevated significantly in this condition and results in reduced levels of NO. Thus, stretch intensity is an important factor in determining ROS balance to ensure healthy cellular function in the vascular system.

### Increased production of reactive oxygen species by pathological stretch

Cells continuously produce ROS as a by-product of normal mitochondrial electron transfer. There are several forms of ROS, such as superoxide anions (O_2_^−^), peroxynitrite anions (ONOO^−^) and hydroxyl radicals (^−^OH) with the most common being hydrogen peroxide (H_2_O_2_) a by-product of superoxide dismutation. At physiological concentrations, these short-lived reactive intermediates are involved in microbial defense, signal transduction and regulation of the cell cycle (Table 1). ROS act as second messengers in signal transduction cascades including those that mediate FAK phosphorylation and are necessary for cell motility and survival [66]. Physiological stretch results in a decrease in superoxide anion production, as Nox4 expression is reduced in HUVECs. In ECs, Nox4-containing NAD(P)H oxidase complexes have been identified as a major source of superoxide anion formation. However, physiological stretch was found to suppress Nox4, increase NO release and reduce ROS formation, suggesting it performs a vasoprotective role [67]. However, increased levels of ROS in pathological stretch can induce pro-atherogenic or pro-inflammatory conditions in HUVECs. Pathological stretch produces excessive O_2_^−^ that can react alone or through the enzyme superoxide dismutase to generate H_2_O_2_ [68]. H_2_O_2_ later activates NFκB and the subsequent transcriptional activity of adhesion molecules such as VCAM-1. This promotes pro-inflammatory activity that leads to atherosclerosis formation over time [69]. In addition, pathological stretch was found to phosphorylate p66^Shc^ in HAEC, which leads to an increase of superoxide anions and a reduction of NO [68]. p66^Shc^ is an adaptor protein that mediates vascular dysfunction in hypertensive mice [70]. Phosphorylation of p66^Shc^ during pathological stretch disturbs the ROS balance as the superoxide anion reacts with NO to generate ONOO^−^ [71]. This results in reduced NO bioavailability and contributes to endothelial dysfunction as ECs lose NO-dependent anti-atherogenic properties [72]. The findings indicate that pathological stretch is associated with excessive ROS production in ECs that can induce oxidative stress and eventually advance cardiovascular disease.

Interestingly, ECs have developed an adaptive mechanism of producing antioxidant enzymes during pathological stretch in HUVECs [71]. For example, the glutathione peroxidase enzyme can degrade accumulating H_2_O_2_ resulting from the reaction of superoxide anion and NO that is involved in pro-inflammatory gene activation, thereby serving as an adaptive mechanism to protect cells from stress conditions. Furthermore, heat shock proteins (such as HSP10) may be upregulated in stretch-induced cells to ensure proper protein folding in response to stress that is thought to cause protein aggregation and misfolding [73]. Thus, the expression of these proteins may serve as the cell’s protection against cellular injury although we suggest that their effect eventually wanes in response to the sustained production of ROS.

### Mechanical stretch promotes inflammation in ECs

In pathological stretch, IL-8 can be secreted from the Weibel–Palade body’s storage granule that was highly exocytosed in HUVECs [74]. In addition, IL-6 secretion has been shown to be mediated by the inflammatory transcription factor NFκB [75]. The activation of NFκB results from the release of ROS to the cytoplasm that then reacts with Cu/Zn superoxide dismutase to form H_2_O_2_ that can subsequently activate NFκβ [70]. Activation of NFκβ also activates adhesion molecules such as the vascular cell adhesion molecule 1 (VCAM-1) and intercellular adhesion molecule 1 (ICAM-1) that are pro-inflammatory. Additionally, application of mechanical stretch on the co-culture system of ECs and SMC showed that CD40 was also highly expressed in HUVECs [76]. These surface molecules exert pro-inflammatory responses by recruiting monocytes or leukocytes, and their high expression predisposes patients to atherosclerotic plaque development. Taken together, evidence of expression of VCAM-1, ICAM-1 and IL-6/8 supports the proposition that mechanical stretch, especially at pathological intensity, stimulates protein expression associated with monocyte, leukocyte and T-cell recruitment and can lead to atherosclerosis as it stimulates the invasion and accumulation of white blood cells in the blood vessels (Table 1).

### Apoptosis and survival of ECs during mechanical stretch

Cyclic stretch affects apoptosis and survival of ECs, and this appears to be based on the magnitude and duration of stretch applied to the cell (Table 1). Apoptosis of ECs is involved in plaque development by inducing inflammatory cell infiltration into the sub-endothelial layer of the vessel wall. In addition, luminal ECs in atherosclerotic plaques that undergo apoptosis are more likely to cause plaque erosion and rupture [45]. Application of physiological stretch on BAEC was found to reduce apoptosis via P13K and Akt activation [45]. This can be explained further by noting that Akt was phosphorylated in 5 % stretch, whereas when 20 % stretch was applied to HAEC, Akt was dephosphorylated [77]. Akt promotes cell survival as it enables the phosphorylation of the pro-apoptotic protein BAD from inducing cell death in BAEC [78]. In addition, expression of NADPH oxidase subunit p22phox in stretched ECs is found to increase ECs survival as p22phox gene knockdown induces pro-apoptotic caspase-3. The discrepancies between physiological and pathological stretch in the promotion of apoptosis indicate that physiological stretch plays a vasculoprotective role whereas pathological stretch plays a role in the promotion of EC apoptosis [77].

## Pathological implications of mechanical stretch

**Fig. 2:**
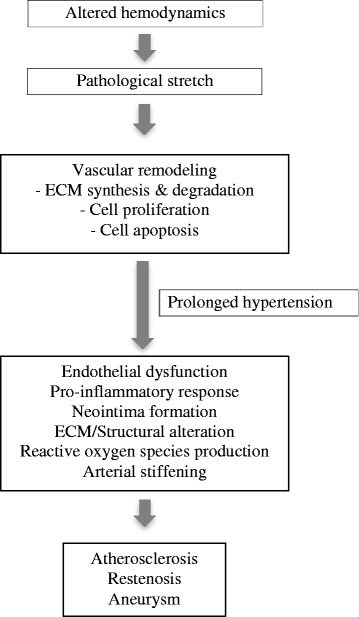
Pathological consequences of altered mechanical stretch. Pathological stretch could change the hemodynamic properties of blood flow in the vascular system. The excessive strain causes cell deformation and the endothelial cell response activates biochemical signaling. Vascular adaptation through remodeling results in ECM synthesis and degradation, proliferation and apoptosis to maintain the vascular physiological state. However, persistent pathological mechanical stretch due to hypertension triggers endothelial dysfunction, pro-inflammatory responses, neointima formation, structural alteration, ROS formation and arterial stiffening. These result in the formation of vascular anomalies such as atherosclerosis, restenosis and aneurysms

**Fig. 3:**
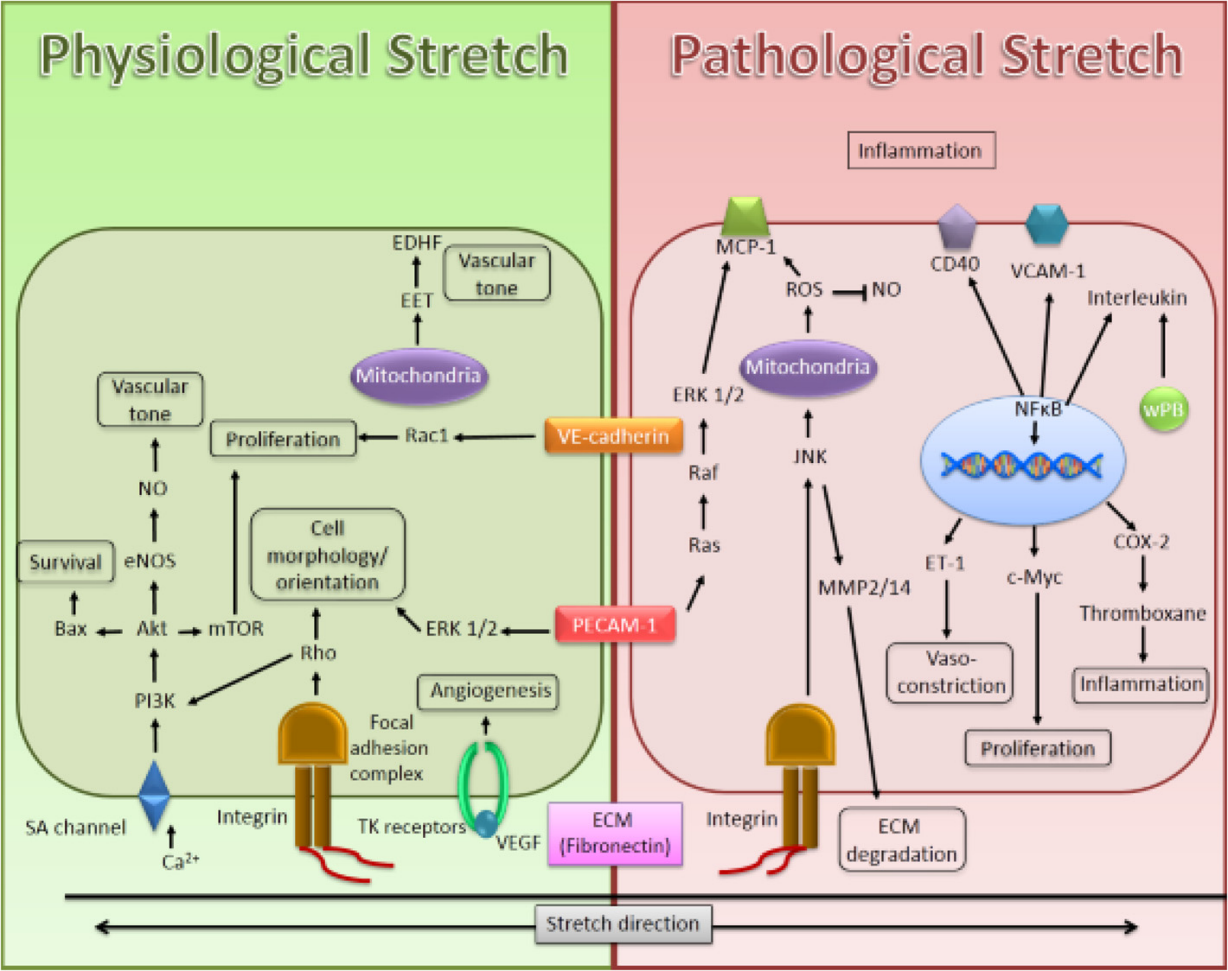
Summary of the mechanisms involved in human cerebral microvascular endothelial cells induced by mechanical stretching. Stretch stimuli are sensed by mechanoreceptors of the endothelial cell that transduce downstream protein signals. This will result in gene activation and increased protein synthesis that alters cell phenotype and function. However, different stretch intensity, magnitude and duration may activate different mechanisms. Physiological stretch is beneficial in maintaining healthy blood vessels; however, pathological stretch, as is observed in hypertension, could activate pathways leading to disease development. Thus, it is important to understand and elucidate the signaling involved with these processes as this could aid in the identification of novel therapeutic approaches aimed at treating vascular related diseases. *Ca* ^*2+*^ Calcium ion, *ECM* Extracellular matrix, *EDHF* Endothelium derived hyperpolarizing factor, *EET* Epoxyeicosatrienoic acid, *eNOS* Endothelial nitric oxide synthase, *ET-1* Endothelin 1, *MCP-1* Monocyte chemoattractant protein-1, *NO* Nitric oxide, *PECAM-1* Platelet endothelial cell adhesion molecule 1, *ROS* Reactive oxygen species, *SA channel* Stretch activated channel, *TK receptors* Tyrosine kinase receptors, *VCAM-1* Vascular cell adhesion molecule-1, *VE-cadherin* Vascular endothelial cadherin, *wPB* Weibel-Palade Bodies

Normal vascular function begins with a mechanical stimulus that becomes converted into a cascade of chemical events to activate protein signaling as a response to the stimuli. As such, ECs are one of the components in blood vessels that are highly organized to sense and respond to normal forces. When unusual conditions arise, such as mechanical overload due to excessive and/or chronic stretch intensity, cells respond with adaptive processes that can become maladaptive and can lead to disease states. As has been mentioned previously, pathological stretch activates different mechanisms leading to significant changes in the phenotype of the cell that may lead to endothelial dysfunction and hence to vascular disease (Figs. 2 and 3).

As high-intensity stretch introduces a stressful environment to the blood vessels, they are modified to accommodate this by a collective process that has been termed ‘vascular remodeling’. This process involves vascular alteration in the form of migration, proliferation, apoptosis and ECM reorganization involving ECM synthesis and degradation [79]. However, degradation of ECM in blood vessels is related to the development of atherosclerosis as a result of smooth muscle cells migrating into the intima layer due to degradation of the internal elastic lamina in the tunica intima and the subsequent initiation of plaque formation [46]. Chronic hypertension, a state associated with prolonged pathological stretch, promotes pro-inflammatory responses by cytokines (IL-8, IL-6) and MCP-1 and results in recruitment of neutrophils and monocytes to the vessel, leading to the development of atherosclerotic plaques [80]. The accumulation of inflammatory cells at the site of inflammation acts as an initial event for fatty streak or atherosclerotic lesion formation and later induces SMC proliferation and migration into the intima layer leading to intimal thickening [81]. Furthermore, mechanical stretch is found to increase EC stiffening which may exacerbate atherosclerosis [82]. Interestingly, trans-differentiation of ECs to SMCs has been observed when stretch is applied to cells. Specifically, SMC marker genes (SM22-α, α-SMA, caldesmon-1, SM MHC and calponin) were increased by stretch, whereas a subsequent reduction in endothelial markers was observed [83, 84]. The presence of SMC markers on EC suggests EC plasticity towards SMC phenotype occurs during mechanical stretch, and this may contribute to the development of atherosclerosis.

As has been mentioned previously, pathological stretch could increase ROS production. This will in turn induce endothelial dysfunction and act as the initial step of atherogenesis. Endothelial dysfunction is an early indicator of atherogenesis that is characterized by reduced NO production that promotes platelet aggregation, thrombus formation and alterations in vasodilation [85]. Excessive ROS production leads to oxidative stress, which in turn leads to oxidation of low-density lipoproteins, the uptake of which by macrophages is easily compared with non-oxidized lipoproteins in the formation of atheroma. Furthermore, ROS can also alter ECs such that they exhibit a pro-inflammatory phenotype characterized by the overexpression of MCP-1 and VCAM-1 [71]. This attracts inflammatory cells, such as white blood cells, and results in the formation of fatty streaks on the tunica intima during atherosclerosis development.

Stenosis is a common vascular pathology characterized by the narrowing of a blood vessel due to atherosclerosis. Stenosis is treated by the use of balloon angioplasty or stents to widen the vessel. Balloon angioplasty reduces the recurrence of restenosis by 40 %, whereas treatment using stents reduces the recurrence of restenosis by 25 % [86]. It is thought that stretching plays a role in this process by increasing cell proliferation and intimal thickening at the vascular graft area after the treatment, although this has yet to be conclusively proven [81, 87]. As previously mentioned, identification of the Egr-1 gene in stretched cells may hold future therapeutic potential as this gene is involved in cell proliferation and silencing it may prevent this process [58].

Another vascular pathology that may be associated with stretch is aneurysm formation. Aneurysms are formed due to the weakening of blood vessels, and their rupture in the brain is considered a cause of strokes. Approximately 2–3.2 % of the general population of the world develops intracranial aneurysms, and the rupture of aneurysms affects approximately 6 per 100,000 people per year [88–90]. Excessive stress could exacerbate the conditions leading to aneurysm rupture as there is a weakening of the vascular structure due to ECM degradation by MMP and cell apoptosis. The rupture of brain aneurysms has recently been reported to be caused by a mechanical force against the thin aneurysm wall [91]. Thus, further study to elucidate mechanical stretch as the etiology for aneurysm development and rupture may assist in understanding aneurysm pathology.

## Future research

The cells of the vascular system are exposed to complex environments and interact with various cell types, hormones, mechanical forces and other vasoactive substances. Due to the complexity of the cellular environment, it is particularly challenging to investigate specific outcomes from mechanical stretch of ECs. Thus, the application of stretch to ECs *per se* has unraveled protein signaling pathways and phenotypic changes as well as pathological consequences. It is therefore not surprising that designing experiments that simulate the conditions that exist in the vascular environment are near impossible. However, a reductionist approach has provided insight into some of mechanisms that can be pieced together to form a fragmented, although detailed, picture.

Shear stress and tensile stretch are two forces that are exerted on the vascular system, but these have contrasting effects on ECs, thus making it challenging to determine the precise mechanisms involved when both stimuli are applied [92]. Therefore, a mechanical device capable of combining forces has been manufactured to explore its simultaneous effect on ECs [93, 92]. In addition, the application of co-culture systems can simulate more accurate complex vascular systems such as those in which ECs have close contact with SMCs. These approaches are still limited, but they may elucidate interactions between ECs and SMCs under conditions of mechanical stress. Outcomes may vary based on differences in stretch frequency, load cycle, amplitude, substrate rigidity and cell confluence [26, 34, 37, 94].

One recent addition to the “omics” suite dubbed “mechanomics” involves generating tools to map global molecular and cellular responses induced by mechanical forces [95]. Application of these technologies could help elucidate comprehensive patterns of expression of genes (genomic), mRNA (transcriptomic), proteins (proteomic) and metabolites (metabolomics); however, the spatio-temporal nature of these technologies may be limiting. These technologies undoubtedly rely on a significant infrastructure and knowledge base, and, therefore, bioinformatics is an invaluable tool in teasing out the mechanistic implications of the protein and gene expression levels. As these fields continue to develop, combinations of gene expression, protein expression, metabolite data and transcriptomic data will provide a comprehensive understanding of stretch biology that will better illustrate the mechanotransduction processes [95].

## Conclusion

Mechanical stretch involves various extracellular to intracellular changes in the coordination of ECs that leads to phenotypic changes in these cells. The response is dynamic such that aberrant changes lead to an imbalance in homeostasis and potentially lead to failure of function or undesired effects that can lead to hypertension and atherosclerosis, among other pathologies. ECs have complex adaptation mechanisms to counteract this stressful state, and a better understanding of these would be helpful in finding novel therapeutic approaches related to hypertension, atherosclerosis, restenosis and aneurysm formation.
